# Early-life parental income mobility and risk of depression and anxiety in adolescence and young adulthood among non-immigrants and second-generation immigrants

**DOI:** 10.1186/s12916-026-05382-x

**Published:** 2026-11-02

**Authors:** Kenta Okuyama, Sara Larsson Lönn, Tsuyoshi Hamano, Ardavan M. Khoshnood, Jan Sundquist, Kristina Sundquist

**Affiliations:** 1 Center for Primary Health Care Research, Department of Clinical Sciences Malmö, Lund University, Malmö, Sweden; 2 University Clinic Primary Care, Skåne University Hospital, Region Skåne, Sweden; 3 Department of Sports Sociology and Health Sciences, Faculty of Sociology, Kyoto Sangyo University, Kyoto, Japan; 4 Emergency Medicine, Department of Clinical Sciences Malmö, Lund University, Skåne University Hospital, Malmö, Sweden

**Keywords:** Anxiety, Depression, Income mobility, Life course, Region of origin, Second-generation immigrant

## Abstract

**Background:**

Little is known about the association between early-life income mobility patterns and the subsequent risk of depression/anxiety among immigrant populations. Understanding these associations may help identify persistent low income and declining income as potential risk factors for depression and anxiety in immigrant populations. We aimed to investigate whether parental income mobility patterns during childhood are associated with the risk of depression/anxiety during adolescence and young adulthood among non-immigrants and second-generation immigrants, and whether the association differs by immigrants’ region of origin.

**Methods:**

Swedish nationwide data on 1,194,015 non-immigrants and 315,793 second-generation immigrants who turned the age of 15 between 2001 and 2020 were used. Income mobility patterns were identified using group-based trajectory modeling based on annual parental income data when the study individuals were aged 0–14. Depression/anxiety was assessed using the National Patient Register (inpatient and outpatient) when the study individuals were aged 15–25. Cox proportional hazard models were used to examine the association between income mobility and depression/anxiety by immigrant status and region of origin.

**Results:**

Five income mobility patterns were identified. Persistent low income was more common among second-generation immigrants than non-immigrants (32.2% versus 12.2%). Compared with persistent high income, persistent low income and downward mobility were associated with higher hazards of depression/anxiety in most groups. Associations were generally weaker among second-generation immigrants, particularly in those from the Middle East or North Africa (MENA). For persistent low income, adjusted hazard ratios (95% Cis) were 2.35 (2.27–2.43) and 2.23 (2.17–2.29) among non-immigrant males and females, versus 1.28 (0.98–1.66) and 1.29 (1.05–1.58) among their MENA counterparts. Among most second-generation immigrant groups, elevated hazards associated with persistent low income were attenuated or absent with upward low–middle mobility.

**Conclusions:**

The association between childhood economic disadvantage and later depression/anxiety varied by immigrant background, with weaker relative risks among second-generation immigrants from MENA. Upward mobility from low to middle income was associated with attenuated risk compared to persistent low income and downward mobility among most second-generation immigrant groups, suggesting that a change of childhood economic circumstances may be relevant to later mental health.

**Supplementary information:**

The online version contains supplementary material available at https://doi.org/10.1186/s12916-026-05382-x.

## Background

The burden of mental disorders has been increasing worldwide [1]. Depression and anxiety are the most common mental disorders, especially during adolescence and young adulthood [1, 2]. Immigrants and their offspring may experience an elevated risk of depression and anxiety due to migration-related and post-migration factors [3]. In addition to these stressors experienced by immigrant families, second-generation immigrants may also be exposed to economic disadvantage, discrimination, challenges related to social integration, and differences in educational and labor market opportunities, all of which may be linked to increased risk of depression and anxiety during adolescence and young adulthood [4–6]. Conversely, some studies have suggested that social and cultural resources, such as strong family cohesion, social support, and religious practices, may contribute to psychological resilience among immigrant populations, and lower risk of depression and anxiety among some immigrant populations has been observed [6–8]. However, previous studies have reported mixed findings regarding mental health among immigrants and their offspring, suggesting that the association between childhood economic disadvantage and later depression and anxiety may differ across immigrant populations.

Among non-immigrant populations, low parental income during childhood has been reported as an important risk factor for depression and anxiety during adolescence and young adulthood [9–11]. Among immigrant populations, on the other hand, the association between parental income and mental disorders has been suggested as being weaker or even absent [11]. A few studies conducted among non-immigrant populations have reported that income mobility patterns measured by longitudinal changes of parental income level during early life was associated with risk of depression, anxiety, schizophrenia, and drug use disorders [10, 12, 13]. These findings are broadly consistent with the social causation hypothesis, whereby economic disadvantage increases the risk of poor mental health through adverse material and psychosocial conditions. However, alternative explanations, including social selection, whereby poor mental health may lead to subsequent economic disadvantage, may also contribute to the observed associations [14, 15]. Unlike income measured at a single point in time or transitions between income categories at a limited number of time points, income mobility patterns characterize the duration, timing, and direction of changes in economic conditions throughout childhood and therefore, it may provide additional insight into whether childhood economic conditions are associated with later mental health.

Little is known, however, about the childhood income mobility patterns among immigrant populations and the association with depression and anxiety during adolescence and young adulthood. Sweden has received a high number of immigrants over the past 50 years; today, one quarter of the population is first- or second-generation immigrants [16]. Identifying income mobility patterns among immigrant populations by region of origin, as well as potentially different associations between income mobility patterns and depression/anxiety among non-immigrants and second-generation immigrants will provide new knowledge.

Given this background, we aimed to investigate whether income mobility patterns during childhood are associated with the risk of depression/anxiety during adolescence and young adulthood among non-immigrants and second-generation immigrants, and whether the association differs by immigrants’ region of origin. Because of the consistent evidence linking childhood economic disadvantage to poorer mental health, we expected that persistent low-income and downward mobility categories would be associated with an increased risk of depression and anxiety and that the associations may differ by immigrants’ region of origin, given the mixed findings regarding mental disorders among immigrant populations.

## Methods

### Study design

Our study used nationwide longitudinal register data covering the entire population of Sweden. Multiple register-based data were linked based on personal identification numbers, which were assigned to all residents in Sweden since birth or immigration. The personal identification numbers were replaced by serial numbers by Statistics Sweden before we had access to the data to ensure the integrity of the participating study individuals. The register data used in our study are described in the appendix (Additional file 1: Table S1). Our study included those who were born between 1995 and 2005. The study individuals were those who were born in Sweden to Swedish-born parents or one/two foreign-born parents. These birth cohorts were selected to ensure complete assessment of parental income mobility patterns throughout childhood (0 to 14 years) while allowing prospective follow-up for the incidence of depression/anxiety from age 15 years onwards within the available period of register data (through 2020). Individuals with depression or anxiety before age 15 years were excluded to ensure that childhood income mobility preceded the onset of the outcome and that only incident depression/anxiety during adolescence and young adulthood was analyzed. Individuals without depression/anxiety during follow-up remained in the cohort until censoring at death, emigration, age 25 years, or the end of follow-up (2020). Individuals with missing information on the covariates were excluded from the analyses. Because the proportion of missing covariate data was very small (approximately 1%), complete-case analysis was considered to be appropriate without losing power and generalizability, and multiple imputation was not performed.

### Outcome

The onset of depression and anxiety was assessed based on the registered physician diagnosis from the National Patient Register, which include inpatient and outpatient records between 2001 and 2020. Depression and anxiety were assessed by the International Classification of Diseases (ICD) code as follows: F32-33 for depression; F40-41, 43 for anxiety, as described in the appendix (Additional file 1: Table S2). They were assessed as a composite outcome (as depression/anxiety) in our primary analysis, as there is a suggested overlap in genetic and environmental factors between the two disorders [17].

### Exposure

Income mobility patterns during childhood and adolescence were assessed based on annual parental income when the study individuals were between the ages of 0–14 years. This age period was chosen to maintain consistency with previous studies [10, 12, 13]. Parental income was based on family disposable income, which is defined as total income, including salary from work, capital, social welfare, and other allowances, subtracted by taxes paid [10, 12, 13]. We applied Group-Based Trajectory Modeling to identify income mobility patterns by utilizing the information on parental income that was recorded every year for each individual from the age of 0 to 14 years. The SAS procedure proc traj was used to estimate five trajectories to represent income mobility patterns in our study population [18]. The selected number of latent trajectory classes was determined based on a combination of statistical model fit (Bayesian Information Criterion [BIC] and Akaike Information Criterion [AIC]), interpretability of the resulting trajectories, and comparability with previous studies. Models with three to six latent trajectory classes were evaluated, and the five-class solution was selected as the most parsimonious and interpretable representation of parental income mobility patterns in the study population. Although a six-class model was also explored, it did not identify an additional distinct downward mobility trajectory but instead subdivided one of the upward mobility trajectories into two trajectories with similar patterns. Because group-based trajectory modeling estimates latent trajectory classes directly from the observed longitudinal data rather than predefined income mobility patterns, the resulting classes were not constrained to be equal in size or symmetrical. Individuals were assigned to the trajectory for which they had the highest posterior probability of membership, and the trajectory labels (e.g., persistent low, downward, upward, and persistent high) were assigned after model estimation to facilitate interpretation.

### Effect modifiers

Immigrant status by region of origin was assessed as an effect-modifying variable of the association between income mobility and depression/anxiety. The study individuals were defined as non-immigrants if they were born in Sweden to two Swedish-born parents. Second-generation immigrants were defined as those who were born in Sweden to at least one foreign-born parent. They were further categorized by six regions of origin based on the country of birth of their parents, i.e., Africa (without North Africa), Asia, Eastern Europe, Latin America and the Caribbean (Latin America), Middle-East or North Africa (MENA), and Western countries. These categories were selected to ensure sufficient sample sizes for subgroup analyses and to maintain comparability with previous Swedish register-based studies using similar regional classifications [19, 20].

### Covariates

Birth year, parental education, parental depression/anxiety, and neighborhood socioeconomic status (SES) were considered as potential confounders. Birth year was included to account for cohort differences in follow-up time and diagnostic practices. Parental education and neighborhood SES were included to account for broader dimensions of childhood socioeconomic circumstances that may be associated with both parental income mobility and later depression/anxiety. Parental depression/anxiety was included to account for familial vulnerability to depression/anxiety and its potential influence on family economic conditions. The covariates were assessed as follows: Parental education and neighborhood SES were assessed when the study individuals were at aged 0, which is prior to the assessment of income mobility. Parental education was based on the highest education level attained by any of the parents and was categorized into three levels (Low: compulsory school, Middle: high school, High: university). Neighborhood SES was based on the neighborhood deprivation index that was derived from the proportion of low education, low-income, unemployment, and social welfare assistance recipients within the Demographic Statistical Areas (DeSO), which is the smallest neighborhood unit factoring in the number of inhabitants, topography, and local infrastructure [21]. Neighborhood SES was categorized into three levels according to the standardized mean and standard deviation (SD) (Low: less than one SD from the mean, Middle: within one SD from the mean, and High: more than one SD from the mean). Parental depression/anxiety was assessed as long as data were available (for study individuals aged 0–14) from the National Patient Register.

### Statistical analysis

We followed the study individuals until the first event of depression or anxiety, death, emigration, age of 25 years, or the end of the study period (2020), whichever came first. To examine the association between different income mobility patterns and depression/anxiety, we applied Cox proportional hazard models, adjusting for birth year, parental education, parental depression/anxiety, and neighborhood socioeconomic status. In a supplementary analysis, we additionally applied Aalen’s additive hazard models to estimate the absolute excess of depression/anxiety cases associated with each income mobility category compared with the reference category (persistent high-income). This approach was used to examine whether the observed associations were consistent on the absolute scale in addition to the relative scale estimated by the Cox proportional hazard models. To examine whether the association between income mobility and depression/anxiety differs by immigrant status and region of origin, interaction terms of income mobility and immigrant status by region of origin were included in Cox proportional hazard models as well as Aalen’s additive hazard models, respectively. Proportional hazards assumptions were assessed using Schoenfeld residuals. Because the incidence of depression and anxiety differs substantially by sex during adolescence and young adulthood, and previous studies have reported sex differences in socioeconomic gradients in health, all analyses were stratified by the study individual’s sex [14, 22]. As a supplementary analysis, depression/anxiety were also assessed as separate outcomes rather than combined. Additionally, we conducted a sensitivity analysis adjusting for family size (number of siblings) as an additional covariate. Specifically, the number of siblings in a family at the age of 14 years (i.e., one child, two siblings, three-four siblings, more than five siblings) was adjusted for in the Cox proportional hazard models. Finally, we conducted a sensitivity analysis adjusting for single-parent family structure. Single-parent family structure was categorized according to the parent(s) with whom the study individual was registered at the age 14 years. Because both family size (number of siblings) and single-parent family structure were measured only at the end of income mobility assessment, they were not included as covariates in the main analyses.

## Results

The individuals who were born in Sweden between 1995 and 2005 with information about their biological parents were 832,581 (51.40%) males and 787,098 (48.60%) females. Of those, 49,014 (5.89%) males and 57,718 (7.33%) females who emigrated, died, developed depression, or anxiety, before the age of 15 years were excluded. Those with missing data on covariates (0.20% of males and 0.21% of females) were also excluded, which resulted in a total of 781,964 males and 727,844 females as the final study sample. Among those, 20.87% of males and 20.96% of females were second-generation immigrants.

**Fig. 1:**
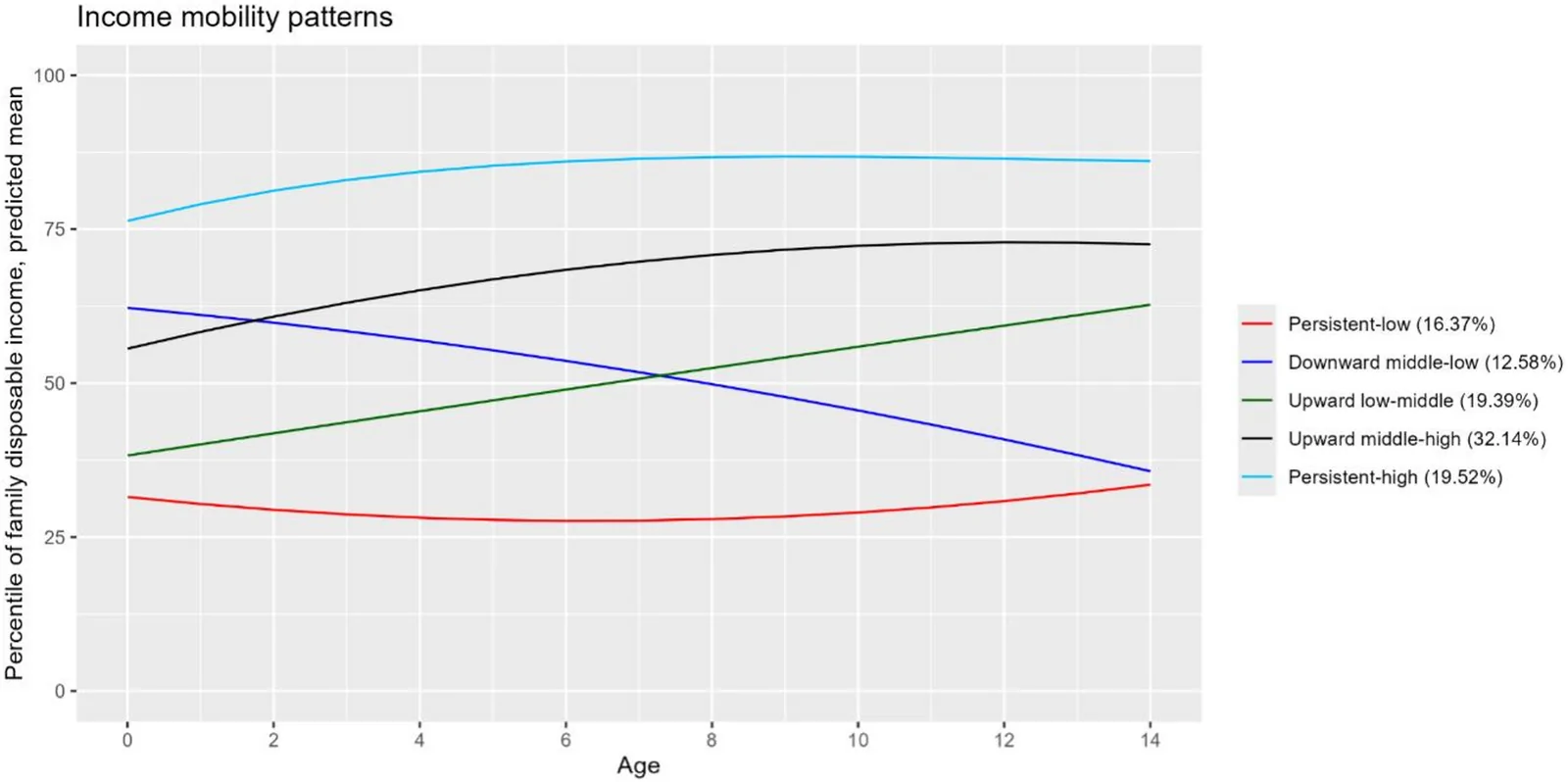
Income mobility patterns identified by group-based trajectory modeling using annual parental income data between the ages of 0–14 years

Five income mobility patterns were identified (Fig. 1). They were classified as persistent low, downward (middle - low), upward (low - middle), upward (middle - high), and persistent high. The proportion of individuals in each income mobility pattern was different between non-immigrants and second-generation immigrants. More than one third of second-generation immigrants were in the persistent low category, whereas 12.15% of non-immigrants were in the persistent low category (Table 1).

**Table 1:** Baseline characteristics of study individuals

	Total	Income mobility				
		Persistent low	Downward (mid–low)	Upward (low–mid)	Upward (mid–high)	Persistent high
Non-immigrants						
N (%)	1,194,015 (79.08%)	145,053 (12.15%)	155,308 (13.01%)	211,074 (17.68%)	421,273 (35.28%)	261,307 (21.88%)
**Birth year**						
Mean (SD)	1996.98 (4.74)	1996.3 (4.6)	1996.65 (4.73)	1996.42 (4.64)	1997.16 (4.74)	1997.7 (4.76)
**Parental education**,** N (%)**						
Low	20,928 (1.75%)	8,226 (5.67%)	3,425 (2.21%)	4,096 (1.94%)	4,054 (0.96%)	1,127 (0.43%)
Middle	254,953 (21.35%)	51,861 (35.75%)	43,889 (28.26%)	56,243 (26.65%)	84,067 (19.96%)	18,893 (7.23%)
High	918,134 (76.89%)	84,966 (58.58%)	107,994 (69.54%)	150,735 (71.41%)	33,3152 (79.08%)	241,287 (92.34%)
**Parental depression/anxiety**,** N (%)**						
Yes	24,341 (2.04%)	7,424 (5.12%)	5,059 (3.26%)	4,214 (2.00%)	5,089 (1.21%)	2,555 (0.98%)
**Neighborhood socioeconomic status**,** N (%)**						
Low	139,693 (11.70%)	34,507 (23.79%)	19,451 (12.52%)	35,463 (16.8%)	40,186 (9.54%)	10,086 (3.86%)
Middle	760,432 (63.69%)	95,589 (65.90%)	105,603 (68.00%)	147,256 (69.77%)	284,637 (67.57%)	127,347 (48.73%)
High	293,890 (24.61%)	14,957 (10.31%)	30,254 (19.48%)	28,355 (13.43%)	96,450 (22.89%)	123,874 (47.41%)
**Second-generation immigrants**						
N (%)	315,793 (20.92%)	101,602 (32.17%)	33,766 (10.69%)	81,422 (25.78%)	65,827 (20.84%)	33,176 (10.51%)
**Birth year**						
Mean (SD)	1997.57 (4.70)	1997.38 (4.62)	1997.03 (4.75)	1997.64 (4.63)	1997.76 (4.78)	1998.14 (4.80)
**Parental education**,** N (%)**						
Low	20,600 (6.52%)	10,147 (9.99%)	2,432 (7.20%)	5,227 (6.42%)	2,242 (3.41%)	552 (1.66%)
Middle	70,927 (22.46%)	29,146 (28.69%)	8,476 (25.10%)	18,710 (22.98%)	12,161 (18.47%)	2,434 (7.34%)
High	224,266 (71.02%)	62,309 (61.33%)	22,858 (67.70%)	57,485 (70.60%)	51,424 (78.12%)	30,190 (91.00%)
**Parental depression/anxiety**,** N (%)**						
Yes	10,004 (3.17%)	4,816 (4.74%)	1,293 (3.83%)	2,178 (2.67%)	1,252 (1.9%)	465 (1.4%)
**Neighborhood socioeconomic status**,** N (%)**						
Low	127,649 (40.42%)	5,5425 (54.55%)	10,275 (30.43%)	40,351 (49.56%)	18,277 (27.77%)	3,321 (10.01%)
Middle	142,770 (45.21%)	39,463 (38.84%)	18,001 (53.31%)	34,220 (42.03%)	35,694 (54.22%)	15,392 (46.39%)
High	45,374 (14.37%)	6,714 (6.61%)	5,490 (16.26%)	6,851 (8.41%)	11,856 (18.01%)	14,463 (43.59%)
**Region of origin**,** N (%)**						
Africa	22,543 (7.14%)	4,478 (10.21%)	4,493 (6.37%)	4,555 (7.06%)	4,545 (4.83%)	4,577 (3.27%)
Asia	48,245 (15.28%)	16,984 (16.72%)	4,644 (13.75%)	13,987 (17.18%)	9,137 (13.88%)	3,493 (10.53%)
Eastern Europe	57,340 (18.16%)	16,448 (16.19%)	5,009 (14.83%)	18,122 (22.26%)	13,177 (20.02%)	4,584 (13.82%)
Latin America and the Caribbean	21,019 (6.66%)	7,822 (7.7%)	2,301 (6.81%)	5,023 (6.17%)	4,136 (6.28%)	1,737 (5.24%)
Middle East or North Africa	66,813 (21.16%)	29,810 (29.34%)	5,372 (15.91%)	20,747 (25.48%)	8,255 (12.54%)	2,629 (7.92%)
Western	99,833 (31.61%)	20,163 (19.85%)	14,290 (42.32%)	17,792 (21.85%)	27,941 (42.45%)	19,647 (59.22%)

The crude incidence rate of depression/anxiety was the highest among second-generation immigrants from Latin America, followed by Western countries, and non-immigrants for both males and females (Table 2). The same pattern was observed when depression and anxiety were assessed separately. The incidence rates of depression/anxiety among females were approximately twice as high as those among males for all non-immigrant and second-generation immigrant groups.

**Table 2:** Incidence rate of depression/anxiety in 100,000 person-years in adolescence and young adulthood, 15–25 years old

	Follow-up time	Depression/anxiety	Depression only	Anxiety only
	Mean (SD)	IR (95% CI)	IR (95% CI)	IR (95% CI)
Males				
Non-immigrants	6.92 (3.36)	924 (915; 933)	562 (555; 569)	597 (590; 604)
*Second-generation immigrants*				
Africa	6.17 (3.39)	677 (618; 741)	387 (342; 435)	461 (413; 514)
Asia	6.26 (3.47)	826 (782; 872)	492 (457; 528)	524 (488; 561)
Eastern Europe	6.41 (3.46)	827 (786; 869)	454 (425; 486)	574 (540; 609)
Latin America	6.27 (3.47)	1382 (1296; 1473)	840 (773; 911)	919 (848; 993)
MENA	6.29 (3.44)	787 (750; 825)	412 (386; 440)	562 (531; 594)
Western	7.09 (3.32)	1140 (1106; 1175)	716 (689; 744)	749 (721; 778)
**Females**				
Non-immigrants	6.69 (3.45)	1795 (1781; 1808)	1048 (1038; 1058)	1336 (1324; 1347)
*Second-generation immigrants*				
Africa	6.00 (3.43)	1233 (1150; 1320)	710 (647; 777)	931 (859; 1008)
Asia	6.07 (3.49)	1513 (1450; 1579)	919 (870; 970)	1086 (1033; 1142)
Eastern Europe	6.29 (3.49)	1509 (1452; 1567)	831 (789; 875)	1144 (1094; 1195)
Latin America	5.98 (3.54)	2658 (2529; 2791)	1521 (1424; 1623)	2074 (1960; 2192)
MENA	6.14 (3.45)	1476 (1423; 1530)	786 (747; 826)	1118 (1072; 1165)
Western	6.79 (3.43)	2189 (2139; 2241)	1320 (1280; 1360)	1689 (1645; 1734)

Latin America: Latin America and the Caribbean. MENA: Middle East or North Africa. IR: Incidence rate. CI: Confidence interval

We observed significantly higher hazards of depression/anxiety among those in the persistent low- income category compared to the persistent high-income category across most groups. However, the association did not reach statistical significance among second-generation immigrant males from Africa or MENA (Table 3). Similarly, significantly higher hazards of depression/anxiety were observed among those in the downward income mobility category compared to the persistent high-income category, except for second-generation immigrant males and females from MENA. Among those in the upward (low–middle) mobility category, the elevated hazards of depression/anxiety relative to the persistent high-income category were substantially attenuated, especially among second-generation immigrants. There was no significant or substantial difference in the hazards of depression/anxiety among those in the upward (middle-high) mobility category compared to the persistent high-income category across all groups. When depression and anxiety were analyzed separately, the overall pattern of associations was broadly similar to the main analyses (Additional file 1: Table S3). Likewise, adjustment for family size yielded results that were consistent with the main analyses (Additional file 1: Table S4). Adjustment for single-parent family structure partly attenuated the associations, although the overall pattern of associations was generally similar to that in the primary analyses (Additional file 1:Table S5).

To assess whether the observed associations were consistent on the absolute to the relative scale, we additionally fitted Aalen’s additive hazard models. The overall pattern of associations was comparable to that of the Cox proportional hazard models, with the greatest excess cases of depression/anxiety observed among individuals in the persistent low-income and downward mobility categories, while this seemed to be less pronounced among second-generation immigrants from MENA. The excess cases for most second-generation immigrants were then substantially attenuated among those in the upward (low-middle) mobility category (Additional file 1: Figure S1). These findings indicate that the main results were robust regardless of whether associations were assessed on the relative or absolute scale.

**Table 3:** Results of Cox proportional hazard model for the association between income mobility patterns and depression/anxiety

	Income mobility				
	Persistent High	Persistent Low	Downward: middle – low	Upward: low – middle	Upward: middle - high
		**HR (95% CI)**	**HR (95% CI)**	**HR (95% CI)**	**HR (95% CI)**
**Males**					
Non-immigrants (*N* = 618,756)	Ref	2.35 (2.27; 2.43)	1.76 (1.70; 1.82)	1.39 (1.34; 1.44)	1.05 (1.02; 1.08)
SG immigrants					
Africa (*N* = 11,478)	Ref	1.50 (0.93; 2.41)	1.86 (1.11; 3.13)	1.02 (0.62; 1.69)	1.03 (0.61; 1.74)
Asia (*N* = 24,946)	Ref	1.86 (1.42; 2.43)	1.87 (1.39; 2.51)	1.35 (1.03; 1.79)	1.26 (0.95; 1.68)
Eastern Europe (*N* = 29,638)	Ref	1.45 (1.18; 1.79)	1.29 (1.02; 1.64)	0.80 (0.64; 0.99)	0.81 (0.65; 1.01)
Latin America (*N* = 10,936)	Ref	1.83 (1.36; 2.46)	1.46 (1.04; 2.05)	1.40 (1.03; 1.91)	1.21 (0.88; 1.66)
MENA (*N* = 34,549)	Ref	1.28 (0.98; 1.66)	1.11 (0.82; 1.50)	0.86 (0.65; 1.13)	0.94 (0.70; 1.26)
Western (*N* = 51,661)	Ref	2.02 (1.82; 2.25)	1.59 (1.42; 1.78)	1.37 (1.22; 1.54)	1.10 (0.99; 1.22)
**Females**					
Non-immigrants (*N* = 575,259)	Ref	2.23 (2.17; 2.29)	1.75 (1.70; 1.79)	1.43 (1.39; 1.47)	1.10 (1.07; 1.13)
SG immigrants					
Africa (*N* = 11,065)	Ref	1.87 (1.29; 2.70)	1.77 (1.18; 2.66)	1.15 (0.78; 1.69)	1.17 (0.78; 1.76)
Asia (*N* = 23,299)	Ref	1.92 (1.57; 2.35)	1.75 (1.40; 2.19)	1.32 (1.08; 1.63)	1.20 (0.97; 1.49)
Eastern Europe (*N* = 27,702)	Ref	1.82 (1.54; 2.16)	1.80 (1.49; 2.16)	1.06 (0.89; 1.26)	1.00 (0.84; 1.19)
Latin America (*N* = 10,083)	Ref	1.85 (1.47; 2.34)	1.69 (1.30; 2.19)	1.38 (1.08; 1.76)	1.23 (0.96; 1.59)
MENA (*N* = 32,264)	Ref	1.29 (1.05; 1.58)	1.23 (0.98; 1.55)	0.92 (0.75; 1.14)	0.93 (0.74; 1.16)
Western (*N* = 48,172)	Ref	1.98 (1.82; 2.15)	1.66 (1.52; 1.81)	1.36 (1.25; 1.49)	1.14 (1.05; 1.23)

SG: Second-generation. Latin America: Latin America and the Caribbean. MENA: Middle East or North Africa. HR: Hazard ratio. CI: Confidence interval. All covariates were adjusted

## Discussion

Using Swedish longitudinal register data, we identified five income mobility patterns during childhood, and second-generation immigrants were markedly in the persistent low-income category. Compared to the persistent high-income group, we found evidence that being in the persistent low-income category and downward (middle – low) mobility category was associated with an increased risk of depression/anxiety during adolescence and young adulthood among non-immigrants and second-generation immigrants, except for those from MENA. The elevated risk of depression/anxiety observed in the persistent low-income and downward mobility category was substantially attenuated or absent in the upward (low – middle) mobility category among most non-immigrant and second-generation immigrant groups. These findings were consistent in the supplementary additive hazard models, indicating that the observed associations were robust when assessed on both relative and absolute scales.

Our study findings are broadly consistent with Danish register studies linking early-life income trajectories with later psychiatric outcomes among non-immigrant populations. For example, one study reported that upward income mobility during childhood was associated with decreased risk of depression and anxiety in adolescence and young adulthood [10]. Another study, albeit on schizophrenia, reported approximately four times increased risk in adolescence and young adulthood among those in the persistent low-income category during childhood compared to those in the persistent high-income category (HR: 4.12, 95% CI: 3.71–4.58) [12]. They also reported that the elevated risk was substantially attenuated by an upward mobility, even from the lowest to the second-lowest income categories (HR: 2.80, 95% CI: 2.46–3.71) [12].

One previously published Swedish register study reported that moving into poverty, i.e., defined as < 60 percentile of parental income, during childhood was associated with increased risk of drug use disorders compared to never poor groups among non-immigrants and second-generation immigrants, and the estimated association was slightly smaller in magnitude among second-generation immigrants (HR: 1.82, 95% CI: 1.66–1.99, and HR: 1.57, 95% CI: 1.34–1.84, respectively) [13]. Our findings also suggested that the associations between the persistent low-income and downward mobility categories and depression/anxiety were generally smaller in magnitude among some second-generation immigrants, especially those from MENA, as reflected by HRs that were closer to null. To our knowledge, few previous studies have examined whether early-life parental income mobility patterns is differently associated with depression and anxiety among non-immigrants and second-generation immigrants by region of origin. Several explanations may account for the observed differential associations. For example, the smaller relative associations observed among second-generation immigrant groups could be explained by potentially protective factors among second-generation immigrants against adverse life circumstances. A number of previous studies suggest higher psychological resilience or adaptation to adverse life circumstances among immigrants compared to non-immigrants, partly because of their cultural and religious norms [8]. However, as individuals in the persistent low-income and downward mobility categories still experienced elevated risks of depression/anxiety, these factors are unlikely to fully offset the adverse consequences of economic disadvantage. Additionally, evidence regarding mental health among immigrant populations remains mixed, with studies reporting both elevated risk of depression/anxiety and evidence consistent with psychological resilience or healthy immigrant effect depending on the population and study settings [3, 6, 8, 23]. Moreover, childhood economic conditions may explain a smaller proportion of the variation in mental health among some immigrant groups if migration-related experiences, discrimination, social support, or other post-migration factors play a comparatively larger role. Therefore, we cannot determine that the observed attenuation of the associations among second-generation immigrants were due to unmeasured risk or protective factors specific to this group [24]. Our data do not allow us to determine the relative contributions of these potentially protective factors and chronic material deprivation, and these mechanisms warrant further investigation. Furthermore, because depression and anxiety were identified through physician-recorded diagnoses, differences in healthcare utilization and access across immigrant groups might have influenced the observed associations. Nonetheless, it is important to note that more than one-third of second-generation immigrants were in the persistent low-income or downward mobility categories, and the smaller relative associations observed should not be interpreted as evidence that economic disadvantage is not important in these groups. One important finding was that the elevated risk of depression/anxiety observed among individuals in the persistent-low income and downward mobility category was substantially attenuated among those who experienced upward mobility from low to middle income during childhood, suggesting that future studies should evaluate whether improvement in childhood economic conditions may be associated with better mental health in adolescence and young adulthood. The lack of a significant difference between the upward (middle–high) mobility and persistent high-income categories suggests that attaining a high-income level during childhood may be protective against depression and anxiety, regardless of timing.

Another notable finding was that, despite females experiencing substantially higher incidence rates of depression and anxiety than males, the HRs were broadly similar across sex in terms of the associations between childhood income mobility patterns and later depression/anxiety. Previous studies among adults have suggested that socioeconomic gradients in health may differ between males and females [14]. In contrast, our findings may indicate that the mechanisms of childhood economic conditions and later mental health operate similarly among males and females during this developmental period.

Potential mechanisms of the association between low-income and depression/anxiety could be chronic psychological stress due to material deprivation within the home and neighborhoods [15]. Because our analyses adjusted for parental education and neighborhood socioeconomic status, the estimated associations should be interpreted as the relationship between parental income mobility and depression/anxiety after accounting for these other dimensions of childhood socioeconomic circumstances. Thus, income mobility in this study captures changes in familial economic conditions during childhood, rather than overall socioeconomic disadvantage. Low-income is also associated with unstable family circumstances, e.g., child neglect and impaired parent-child relationships [25], as well as low academic performance and educational attainment [26], which may all increase the risk of depression/anxiety in later life [27]. In the sensitivity analysis, adjustment for single-parent family structure at age 14 partly attenuated some of the associations, suggesting that it may have contributed to the associations between parental income mobility and later depression/anxiety to a certain extent. However, because single-parent family structure only was assessed at the end of the study period, its role as a confounding or mediating factor cannot be distinguished in our data. Future studies could investigate the potentially different mechanisms of the association between income mobility and depression/anxiety among second-generation immigrants by region of origin.

### Implications

Previous studies have suggested that financial support to households with financial difficulties is effective in reducing the risk of mental health outcomes among children [28, 29]. However, given the attenuated association between income mobility and depression/anxiety among second-generation immigrants, as well as their high representation in the persistent low-income category, further investigations on underlying factors are needed. It is still important to note that more than one-third of second-generation immigrants were in the persistent low-income or downward mobility categories, and they may benefit from upward mobility.

Although our study was conducted in Sweden, the findings may be informative for other high-income countries with comparable welfare systems and immigrant populations, particularly those in Scandinavia and parts of Europe. However, differences in immigration histories, social welfare policies, healthcare systems, and socioeconomic inequalities may influence the magnitude and direction of these associations. Therefore, replication in other settings, including countries with different healthcare and social policy contexts, is needed to assess the broader generalizability of our findings.

### Strengths

Our study assessed early-life income mobility by group-based trajectory modeling based on annual parental income data from the high-quality nationwide Swedish register data [30]. The longitudinal nature and multiple register data allowed us to ensure temporality between exposure, outcome, and potential confounders. The register data also covered the entire population, so that selection bias was minimized.

### Limitations

One of the limitations in our study was that we could not claim causality in the association between income mobility and depression/anxiety. Nevertheless, our findings are robust by assessing parental income and its mobility based on yearly data from 0 to 14 years of age, compared to previous studies that assessed income by a single or only a few time points [11, 31]. Another limitation is that the income mobility patterns were identified using a latent class approach. Although the number of trajectories was selected based on statistical fit and interpretability, alternative trajectory groups or labeling strategies may also be plausible explanations behind potential associations. Therefore, the trajectory groups should be regarded as descriptive summaries of the estimated income mobility patterns rather than universally applicable income mobility categories. With respect to the outcome, depression and anxiety were assessed from age 15 to 25 years, a developmental period when common mental disorders emerge and contribute substantially to disease burden [2, 32]. International studies, including Global Burden of Disease analyses, indicate that depression and anxiety are major contributors to the mental health burden among adolescents and young adults [2, 32]. However, direct comparison with our estimates should be made cautiously because our outcome was based on diagnoses recorded in Swedish inpatient and outpatient registers, whereas many international estimates are based on self-reported symptoms, survey-based prevalence, or modeled burden estimates. Therefore, differences in healthcare utilization, diagnostic practices, and outcome assessment should be considered when comparing our findings with those from other settings. Additionally, register-based assessment of depression and anxiety might have been affected by potentially different healthcare-seeking behaviors across non-immigrants and second-generation immigrants from different regions [33]. Nevertheless, the observed associations between childhood income mobility and mental health may be more broadly informative given the consistency with previous findings. The regions of origin used in this study were quite broad and may mask heterogeneity within each group with respect to migration history, socioeconomic position, and culture. Therefore, differences by region of origin should be interpreted cautiously as population-level patterns across broad geographic groupings rather than as evidence of homogeneous effects within these regions. Finally, depression and anxiety are both known to be hereditary, and therefore, we adjusted for parental depression and anxiety as they also may contribute to income mobility patterns. This adjustment could reduce but not fully eliminate the possibility that children’s depression/anxiety are due to parental depression/anxiety, which could in turn explain some of the income mobility patterns.

## Conclusions

Our findings indicate that second-generation immigrants were disproportionately represented in the persistent low- income or downward mobility categories throughout childhood and adolescence, and that was associated with increased risk of depression/anxiety in young adulthood. Upward mobility from the low to the middle category attenuated the increased risk of depression/anxiety, suggesting beneficial effect of upward social mobility.

## Supplementary information

Below is the link to the electronic supplementary material.

Supplementary Material 1: Additional file 1: Supplementary tables and figure. Table S1: Swedish register data used in the study. Table S2: Register-based definitions and diagnostic codes for depression and anxiety. Figure S1: Associations between childhood parental income mobility patterns and depression/anxiety estimated using Aalen’s additive hazard models. Table S3: Associations between childhood parental income mobility patterns and depression and anxiety analyzed separately using Cox proportional hazards models. Table S4: Associations between childhood parental income mobility patterns and depression/anxiety with additional adjustment for family size. Table S5: Associations between childhood parental income mobility patterns and depression/anxiety with additional adjustment for single-parent family structure.

## Data Availability

The data used in this study are based on Swedish national register data and cannot be made publicly available because of legal and ethical restrictions under Swedish data protection legislation and agreements with the data providers. Researchers who meet the criteria for access to confidential data may apply for permission to use the data from the relevant Swedish authorities (e.g., Statistics Sweden and the Swedish National Board of Health and Welfare) after ethical approval and compliance with the required legal agreements. The authors are not permitted to share the data directly. Access is granted only to the individual researcher and is subject to the conditions specified by the respective data providers and applicable Swedish legislation. Further information regarding the register data can be found, for example, in the Swedish National Board of Health and Welfare (https://www.socialstyrelsen.se/en/statistics-and-data/registers/).

## Abbreviations

AIC: Akaike information criterion
BIC: Bayesian information criterion
CI: Confidence interval
ERC: European Research Council
HR: Hazard ratio
ICD: International Classification of Diseases
IR: Incidence rate
MENA: Middle East or North Africa
SD: Standard deviation
SES: Socioeconomic status
SG: Second-generation

## Abbreviations

AIC: Akaike information criterion
BIC: Bayesian information criterion
CI: Confidence interval
ERC: European Research Council
HR: Hazard ratio
ICD: International Classification of Diseases
IR: Incidence rate
MENA: Middle East or North Africa
SD: Standard deviation
SES: Socioeconomic status
SG: Second-generation
