# Supplementary material for: Early-life parental income mobility and risk of depression and anxiety in adolescence and young adulthood among non-immigrants and second-generation immigrants
Source: BMC Med. 2026 Nov 2;24:612. doi: 10.1186/s12916-026-05382-x (PMC13729354; doi:10.1186/s12916-026-05382-x)
Supplement: Supplementary file 1 [file 12916_2026_5382_MOESM1_ESM.docx]

**Supplementary appendix**

[Table S1: Swedish register data used in the study	2](#_Toc238791033)

[Table S2: Definitions of depression and anxiety by register and their identification codes that are used in this study	3](#_Toc238791034)

[Figure S1: Results of Aelan’s additive hazard model for the association between income mobility patterns and depression/anxiety.	4](#_Toc238791035)

[Table S3: Results of Cox proportional hazard model for the association between income mobility patterns and depression, anxiety, respectively.	5](#_Toc238791036)

[Table S4: Results of Cox proportional hazard model for the association between income mobility patterns and depression/anxiety also adjusting for family size.	6](#_Toc238791037)

[Table S5: Results of Cox proportional hazard model for the association between income mobility patterns and depression/anxiety also adjusting for single-parent	7](#_Toc238791038)

# **Table S1: Swedish register data used in the study**

| **Data** | **Available year of data and Description** | **Assessed variables** |
| --- | --- | --- |
| Multi-Generation Register | 1932-2020.  Linked children and parents who were born in Sweden or immigrated to Sweden together with parents after 1932. | Immigrant status.  Region of origin. |
| Total Population Register | 1968-2020.  Sociodemographic information. | Sex.  Age.  Immigrant status.  Region of origin.  Emigration. |
| National Patient Register |  |  |
| Inpatient records | 1964-2020.  Hospitalization. | Depression.  Anxiety. |
| Outpatient records | 2001-2020.  Hospital visits. | Depression.  Anxiety. |
| Longitudinal Integration Database for Health Insurance and Labor Market Studies (LISA) | 1990-2020.  Socioeconomic information. | Parental income.  Parental education.  Neighborhood socioeconomic status. |
| Cause of Death Register | 1961-2020.  Information of all deaths. | To censor individuals. |

# **Table S2: Definitions of depression and anxiety by register and their identification codes that are used in this study**

| **Depression** | |
| --- | --- |
| Inpatient and Outpatient records | |
| *ICD-9** | |
| Maniac-depressive psychosis, depressed type  Depressive type psychosis  Neurotic depression | 296.1  298.0  300.4 |
| *ICD-10* |  |
| Depressive episode  Recurrent depressive disorder | F32, 33 |
| **Anxiety** |  |
| Inpatient and Outpatient records | |
| *ICD-9** | |
| Anxiety states  Phobic States | 300.0  300.2 |
| *ICD-10* | |
| Phobic anxiety disorders  Other anxiety disorders  Reaction to severe stress, and adjustment disorders | F40, 41, 43 |

ICD: International Classification of Diseases. *ICD-9 codes were used for parental depression and anxiety when records predated the adoption of ICD-10.

# **Figure S1: Results of Aalen’s additive hazard model for the association between income mobility patterns and depression/anxiety.**

# **Table S3: Results of Cox proportional hazard model for the association between income mobility patterns and depression, anxiety, respectively.**

|  | **Income mobility** | | | | |
| --- | --- | --- | --- | --- | --- |
|  | Persistent High | Persistent  Low | Downward:  middle – low | Upward:  low – middle | Upward:  middle - high |
|  |  | **HR (95% CI)** | **HR (95% CI)** | **HR (95% CI)** | **HR (95% CI)** |
| **Depression** |  |  |  |  |  |
| **Males** |  |  |  |  |  |
| Non-immigrants | Ref | 2.29 (2.19; 2.39) | 1.73 (1.66; 1.81) | 1.42 (1.36; 1.48) | 1.06 (1.02; 1.11) |
| SG immigrants |  |  |  |  |  |
| Africa | Ref | 1.67 (0.87; 3.20) | 1.95 (0.96; 3.95) | 1.10 (0.55; 2.19) | 1.13 (0.55; 2.32) |
| Asia | Ref | 1.57 (1.14; 2.18) | 1.97 (1.39; 2.80) | 1.21 (0.87; 1.69) | 1.16 (0.82; 1.64) |
| Eastern Europe | Ref | 1.51 (1.13; 2.01) | 1.55 (1.13; 2.14) | 0.87 (0.64; 1.17) | 0.91 (0.68; 1.24) |
| Latin America | Ref | 2.16 (1.45; 3.24) | 1.85 (1.18; 2.90) | 1.41 (0.92; 2.15) | 1.45 (0.95; 2.22) |
| MENA | Ref | 1.19 (0.84; 1.69) | 1.05 (0.70; 1.56) | 0.78 (0.54; 1.12) | 0.85 (0.58; 1.26) |
| Western | Ref | 1.83 (1.60; 2.09) | 1.5 (1.30; 1.73) | 1.35 (1.17; 1.55) | 1.05 (0.92; 1.20) |
| **Females** |  |  |  |  |  |
| Non-immigrants | Ref | 2.07 (2.00; 2.15) | 1.70 (1.64; 1.76) | 1.36 (1.32; 1.41) | 1.08 (1.05; 1.12) |
| SG immigrants |  |  |  |  |  |
| Africa | Ref | 1.83 (1.12; 2.98) | 1.98 (1.16; 3.37) | 1.08 (0.64; 1.81) | 1.29 (0.76; 2.20) |
| Asia | Ref | 1.98 (1.52; 2.58) | 2.01 (1.50; 2.69) | 1.39 (1.06; 1.83) | 1.33 (1.00; 1.76) |
| Eastern Europe | Ref | 1.71 (1.37; 2.14) | 1.71 (1.33; 2.18) | 1.00 (0.80; 1.26) | 0.96 (0.76; 1.21) |
| Latin America | Ref | 1.31 (0.99; 1.74) | 1.16 (0.84; 1.61) | 1.05 (0.78; 1.41) | 0.88 (0.65; 1.20) |
| MENA | Ref | 1.11 (0.85; 1.44) | 0.90 (0.66; 1.21) | 0.72 (0.55; 0.95) | 0.78 (0.59; 1.04) |
| Western | Ref | 1.88 (1.69; 2.09) | 1.65 (1.48; 1.84) | 1.29 (1.15; 1.44) | 1.12 (1.01; 1.25) |
|  |  |  |  |  |  |
| **Anxiety** |  |  |  |  |  |
| **Males** |  |  |  |  |  |
| Non-immigrants | Ref | 2.35 (2.25; 2.46) | 1.77 (1.69; 1.85) | 1.37 (1.31; 1.43) | 1.04 (1.00; 1.08) |
| SG immigrants |  |  |  |  |  |
| Africa | Ref | 1.67 (0.92; 3.05) | 2.06 (1.08; 3.92) | 1.22 (0.65; 2.27) | 1.08 (0.56; 2.09) |
| Asia | Ref | 2.13 (1.48; 3.06) | 2.00 (1.34; 2.96) | 1.44 (0.99; 2.09) | 1.47 (1.01; 2.16) |
| Eastern Europe | Ref | 1.57 (1.22; 2.01) | 1.15 (0.85; 1.55) | 0.81 (0.63; 1.06) | 0.80 (0.61; 1.04) |
| Latin America | Ref | 1.81 (1.25; 2.63) | 1.46 (0.95; 2.23) | 1.55 (1.06; 2.28) | 1.27 (0.86; 1.89) |
| MENA | Ref | 1.43 (1.04; 1.98) | 1.19 (0.83; 1.72) | 0.91 (0.65; 1.27) | 1.06 (0.74; 1.51) |
| Western | Ref | 2.17 (1.90; 2.48) | 1.71 (1.48; 1.97) | 1.43 (1.24; 1.65) | 1.16 (1.01; 1.32) |
| **Females** |  |  |  |  |  |
| Non-immigrants | Ref | 2.33 (2.26; 2.4) | 1.80 (1.75; 1.86) | 1.48 (1.43; 1.52) | 1.10 (1.07; 1.13) |
| SG immigrants |  |  |  |  |  |
| Africa | Ref | 1.81 (1.19; 2.74) | 1.73 (1.09; 2.75) | 1.13 (0.73; 1.75) | 1.12 (0.71; 1.78) |
| Asia | Ref | 1.85 (1.46; 2.35) | 1.77 (1.36; 2.31) | 1.30 (1.02; 1.66) | 1.22 (0.95; 1.56) |
| Eastern Europe | Ref | 1.89 (1.55; 2.30) | 1.85 (1.48; 2.30) | 1.08 (0.88; 1.32) | 1.03 (0.84; 1.27) |
| Latin America | Ref | 2.02 (1.55; 2.65) | 1.78 (1.32; 2.40) | 1.41 (1.06; 1.87) | 1.35 (1.01; 1.80) |
| MENA | Ref | 1.31 (1.04; 1.67) | 1.27 (0.98; 1.66) | 0.98 (0.77; 1.25) | 0.93 (0.72; 1.21) |
| Western | Ref | 2.01 (1.83; 2.21) | 1.61 (1.46; 1.78) | 1.40 (1.27; 1.55) | 1.15 (1.05; 1.26) |

SG: Second-generation. Latin America: Latin America and the Caribbean. MENA: Middle East or North Africa. HR: Hazard ratio. CI: Confidence interval. All covariates were adjusted for.

# **Table S4: Results of Cox proportional hazard model for the association between income mobility patterns and depression/anxiety also adjusting for family size.**

|  | **Income mobility** | | | | |
| --- | --- | --- | --- | --- | --- |
|  | Persistent High | Persistent  Low | Downward:  middle – low | Upward:  low – middle | Upward:  middle - high |
|  |  | **HR (95% CI)** | **HR (95% CI)** | **HR (95% CI)** | **HR (95% CI)** |
| **Males** |  |  |  |  |  |
| Non-immigrants | Ref | 2.23 (2.16; 2.31) | 1.71 (1.65; 1.77) | 1.37 (1.32; 1.42) | 1.06 (1.03; 1.10) |
| SG immigrants |  |  |  |  |  |
| Africa | Ref | 1.52 (0.94; 2.46) | 1.96 (1.16; 3.29) | 1.02 (0.62; 1.70) | 1.02 (0.60; 1.73) |
| Asia | Ref | 1.80 (1.37; 2.35) | 1.79 (1.33; 2.41) | 1.35 (1.02; 1.77) | 1.27 (0.95; 1.69) |
| Eastern Europe | Ref | 1.41 (1.15; 1.74) | 1.26 (0.99; 1.60) | 0.80 (0.64; 0.99) | 0.81 (0.65; 1.02) |
| Latin America | Ref | 1.75 (1.30; 2.36) | 1.42 (1.01; 1.99) | 1.38 (1.02; 1.89) | 1.21 (0.88; 1.66) |
| MENA | Ref | 1.26 (0.97; 1.64) | 1.08 (0.80; 1.46) | 0.86 (0.66; 1.13) | 0.94 (0.70; 1.25) |
| Western | Ref | 1.98 (1.78; 2.20) | 1.55 (1.39; 1.74) | 1.36 (1.22; 1.52) | 1.11 (0.99; 1.23) |
| **Females** |  |  |  |  |  |
| Non-immigrants | Ref | 2.11 (2.06; 2.17) | 1.69 (1.64; 1.74) | 1.40 (1.37; 1.44) | 1.11 (1.09; 1.14) |
| SG immigrants |  |  |  |  |  |
| Africa | Ref | 1.91 (1.32; 2.75) | 1.82 (1.21; 2.73) | 1.17 (0.79; 1.72) | 1.18 (0.79; 1.77) |
| Asia | Ref | 1.88 (1.54; 2.30) | 1.70 (1.36; 2.13) | 1.31 (1.07; 1.61) | 1.20 (0.97; 1.49) |
| Eastern Europe | Ref | 1.78 (1.51; 2.11) | 1.76 (1.46; 2.13) | 1.06 (0.89; 1.26) | 1.00 (0.84; 1.20) |
| Latin America | Ref | 1.83 (1.45; 2.31) | 1.66 (1.28; 2.15) | 1.36 (1.07; 1.74) | 1.23 (0.95; 1.57) |
| MENA | Ref | 1.30 (1.06; 1.59) | 1.22 (0.97; 1.54) | 0.94 (0.76; 1.16) | 0.94 (0.75; 1.17) |
| Western | Ref | 1.93 (1.78; 2.10) | 1.62 (1.49; 1.77) | 1.35 (1.24; 1.47) | 1.15 (1.06; 1.25) |

SG: Second-generation. Latin America: Latin America and the Caribbean. MENA: Middle East or North Africa. HR: Hazard ratio. CI: Confidence interval. All covariates in the main analyses + family size (number of siblings) were adjusted for.

# **Table S5: Results of Cox proportional hazard model for the association between income mobility patterns and depression/anxiety also adjusting for single-parent**

|  | **Income mobility** | | | | |
| --- | --- | --- | --- | --- | --- |
|  | Persistent High | Persistent  Low | Downward:  middle – low | Upward:  low – middle | Upward:  middle - high |
|  |  | **HR (95% CI)** | **HR (95% CI)** | **HR (95% CI)** | **HR (95% CI)** |
| **Males** |  |  |  |  |  |
| Non-immigrants | Ref | 1.61 (1.55; 1.68) | 1.22 (1.18; 1.28) | 1.24 (1.20; 1.29) | 1.02 (0.99; 1.05) |
| SG immigrants |  |  |  |  |  |
| Africa | Ref | 1.08 (0.65; 1.81) | 1.38 (0.80; 2.38) | 0.95 (0.57; 1.57) | 1.01 (0.59; 1.71) |
| Asia | Ref | 1.23 (0.92; 1.62) | 1.24 (0.91; 1.68) | 1.22 (0.92; 1.61) | 1.21 (0.91; 1.62) |
| Eastern Europe | Ref | 0.94 (0.75; 1.18) | 0.86 (0.67; 1.11) | 0.75 (0.60; 0.93) | 0.79 (0.64; 0.99) |
| Latin America | Ref | 1.25 (0.90; 1.73) | 1.01 (0.70; 1.46) | 1.24 (0.91; 1.70) | 1.16 (0.84; 1.60) |
| MENA | Ref | 0.91 (0.70; 1.20) | 0.79 (0.58; 1.08) | 0.81 (0.61; 1.06) | 0.90 (0.67; 1.21) |
| Western | Ref | 1.48 (1.31; 1.67) | 1.17 (1.03; 1.33) | 1.23 (1.10; 1.38) | 1.07 (0.96; 1.19) |
| **Females** |  |  |  |  |  |
| Non-immigrants | Ref | 1.60 (1.55; 1.65) | 1.27 (1.23; 1.31) | 1.29 (1.26; 1.32) | 1.07 (1.05; 1.1) |
| SG immigrants |  |  |  |  |  |
| Africa | Ref | 1.24 (0.83; 1.84) | 1.20 (0.78; 1.85) | 1.01 (0.68; 1.49) | 1.12 (0.74; 1.68) |
| Asia | Ref | 1.22 (0.99; 1.51) | 1.10 (0.87; 1.39) | 1.18 (0.96; 1.45) | 1.14 (0.92; 1.41) |
| Eastern Europe | Ref | 1.24 (1.03; 1.49) | 1.23 (1.01; 1.50) | 1.01 (0.85; 1.20) | 0.98 (0.82; 1.17) |
| Latin America | Ref | 1.43 (1.11; 1.85) | 1.33 (1.01; 1.76) | 1.26 (0.98; 1.61) | 1.20 (0.94; 1.55) |
| MENA | Ref | 0.92 (0.74; 1.13) | 0.87 (0.69; 1.10) | 0.87 (0.71; 1.08) | 0.91 (0.72; 1.13) |
| Western | Ref | 1.50 (1.37; 1.64) | 1.27 (1.15; 1.40) | 1.24 (1.14; 1.36) | 1.11 (1.02; 1.20) |

SG: Second-generation. Latin America: Latin America and the Caribbean. MENA: Middle East or North Africa. HR: Hazard ratio. CI: Confidence interval. All covariates in the main analyses + single parent were adjusted for.
